# PDMS/PVDF Electrospinning Membranes for Water-in-Oil Emulsion Separation and UV Protection

**DOI:** 10.3390/biomimetics7040217

**Published:** 2022-11-29

**Authors:** Jie Li, Yushan Li, Yiyi Lu, Wentian Shi, Huafeng Tian

**Affiliations:** 1 School of Artificial Intelligence, Beijing Technology and Business University, Beijing 100048, China; 2 Beijing Key Laboratory of Quality Evaluation Technology for Hygiene and Safety of Plastics, School of Chemistry and Materials Engineering, Beijing Technology and Business University, Beijing 100048, China

**Keywords:** electrospinning, polydimethylsiloxane, polyvinylidene difluoride, oil–water separation, UV protection

## Abstract

With industry development, the separation of oily wastewater is becoming more critical. Inspired by organisms such as lotus leaves, biomimetic superhydrophobic surfaces with micro-nano structures have shown great potential in this regard. In this work, PDMS/PVDF oil–water separation membranes with designed microstructures were prepared by electrospinning technology. The membrane-forming effect of electrospinning with different ratios of PDMS and PVDF was studied. The study found that membranes with high PDMS content were more likely to form microspheres, and PDMS tended to concentrate on the microspheres. The results also showed that the microspheres would bring better hydrophobicity to the membrane. When the ratio of PDMS to PVDF is 1:2, the membrane has a water contact angle of up to 150° and an oil contact angle of 0°. At this ratio, the separation efficiency of the membrane for the water-in-oil emulsion is 98.7%, and it can still maintain more than 98% after ten separation cycles, which is a good candidate for oil–water separation. Furthermore, microspheres enable the membrane to achieve macroscopic uniformity and microscopic phase separation so that the membranes have both good elongation and fracture strength. In addition, the PDMS/PVDF membranes also exhibit excellent UV resistance, and their UV protection factor is greater than 185, making them a potential UV protective material.

## 1. Introduction

Crude oil is one of the essential raw materials in modern industrial society, and crude oil products are closely related to our lives. However, in recent years, the frequent oil spill accidents and the discharge of oily industrial sewage have brought catastrophic damage to the environment [1]. Therefore, how to separate oil and water, even the separation of oil and water emulsions has become one of the problems to be solved urgently [2]. Traditional oil–water separation technologies, such as gravity separation and centrifugal separation, are facing problems, such as low separation rate, high cost, and secondary pollution [3]. Inspired by nature, special wettable materials with biomimetic structures provide a new direction for oil–water separation. Among them, membrane separation technology has gradually become the mainstream. In addition, the large pore size of traditional membranes is not suitable for separating oil–water emulsions [4], and it is imperative to prepare separation membranes with high porosity and small pore size. Phase separation [5] and electrospinning [6] are often used to prepare separation membranes with high porosity and small pore size. Compared with phase separation, nanofibrous membranes prepared by electrospinning are more attractive [7]. It has broad application prospects in the fields of biological medicine [8], sewage treatment [9], membrane distillation [10], oil–water separation [11], and other fields. 

With in-depth research on animals and plants such as lotus leaves, butterflies, and nepenthes, scholars have found that special wettability can be imparted to materials by constructing biomimetic surfaces with different micro-nano structures. In addition, the special wettability of the separation membrane is the key to obtaining the oil–water separation capability [12,13]. Among them, materials with both hydrophobicity and lipophilicity can selectively absorb the oil phase in the oil–water mixture, while materials with hydrophilic and oleophobic properties can separate water from oil [14,15,16]. Compared with hydrophilic materials, the theory and preparation basis of hydrophobic materials are more complete [17]. The microstructure is one of the critical factors affecting hydrophobicity, and constructing a reasonable microstructure is the key to the preparation of hydrophobic materials [18]. The porous and multi-layered structure brought by electrospinning technology can trap air and obtain excellent hydrophobic properties by realizing a stable Cassie state [19].

Polydimethylsiloxane (PDMS) has low surface energy and good thermal stability [20], making it a good choice for the preparation of hydrophobic materials [21]. However, PDMS is difficult to electrospin due to its low glass transition temperature (T_g_) [22], so other spinning materials must be added. Commonly used assisted spinning materials to include nanomaterials (silver nanowires [23], carbon nanotubes [24], etc.) and polymers (polyvinyl alcohol [25], polystyrene [26], etc.). Nanomaterials are prone to agglomeration due to their high specific surface area [27] and are not easily dispersed in PDMS uniformly, so polymers are more suitable for electrospinning PDMS. However, PDMS has hydrophobicity, making it challenging to blend hydrophilic polymers with it, so it is more suitable to choose hydrophobic polymers as spinning aid materials. Polyvinylidene difluoride (PVDF) among hydrophobic polymers has good spinnability [28,29] and is an excellent choice for improving the electrospinning of PDMS. In general, polymer blends have better properties than single components [30], and PDMS/PVDF blends have broad application prospects in separation and recovery [31], flexible electronics [32], anti-icing [33] and other fields.

In fact, PDMS and PVDF are mutually incompatible [34], and it is difficult to find a single solvent that can dissolve both PDMS and PVDF. Currently, PDMS/PVDF-based electrospinning membranes are prepared by modified PDMS [35], a sandwich method [36], or a coaxial electrospinning method [37]. That is to say, the PDMS/PVDF electrospinning membrane prepared by the above method has problems such as high cost, complicated processes, and uneven distribution of PDMS. 

In this work, PDMS/PVDF electrospinning membranes were prepared in one step by dissolving PDMS and PVDF in a composite solvent. This approach shortens the preparation time and process and can produce membranes with excellent hydrophobicity in just a few hours. In addition, the study also discussed for the first time the effect of the ratio of PDMS to PVDF on the morphology and performance of electrospinning membranes and found that the optimal ratio was 1:2. Experiments found that the membranes with microspheres showed better hydrophobicity than those without microspheres and could better separate the water-in-oil emulsions. In addition, due to the addition of PVDF, the transmittance of the membrane to UV was significantly reduced, showing excellent shielding ability against UV. The PDMS/PVDF membrane prepared by this method had a short preparation time and a simple process and had a good application prospect.

## 2. Experimental

### 2.1. Materials

PVDF (M_w_ = 400,000) was purchased from Shanghai Aladdin Biochemical Technology Co., Ltd. PDMS prepolymer (SYLGARD 184A) and the curing agent (SYLGARD 184B) was purchased from Changzhou Microflu Technology Co., Ltd. N, N-Dimethylformamide (DMF) (≥99%), tetrahydrofuran (THF) (≥99%), and span80 were purchased from Beijing Innochem Technology Co., Ltd. N-hexane (≥97%) was purchased from Shanghai Mcklin Biochemical Co., Ltd. Oil red was purchased from Beijing Jinming Biotechnology Co., Ltd. Methylene blue was purchased from Tianjin Fuchen Chemical Reagent Co., Ltd. Deionized water was made in the laboratory. All chemical reagents are of analytical grade.

### 2.2. Preparation of PVDF/PDMS Electrospinning Membrane

**Figure 1:**
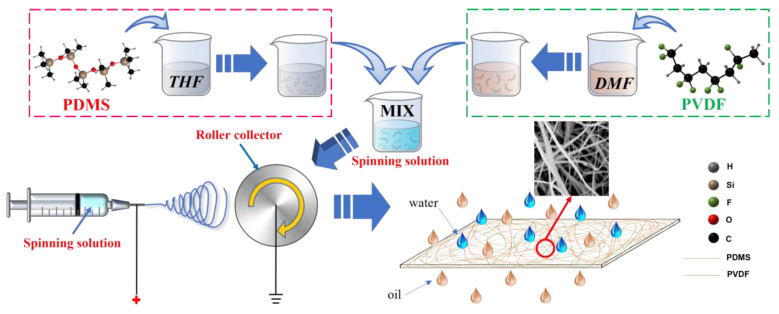
The preparation process of PVDF/PDMS electrospinning membranes.

The preparation process of the PVDF/PDMS electrospinning membrane is shown in Figure 1. First, a certain amount of PVDF was charged into DMF and stirred in a water bath for two hours at 80 °C to prepare PVDF solutions with concentrations of 30%, 25%, 20%, 15%, and 10%, respectively. Then, the PDMS prepolymer and the curing agent were added to THF at a ratio of 10:1 and magnetically stirred for 1 h to obtain a 10% PDMS solution. Finally, PVDF solutions of different concentrations were blended with PDMS solutions in a ratio of 1:1. After stirring at 50 °C for one hour, the spinning solutions with mass ratios of PVDF and PDMS of 3:1, 5:2, 2:1, 3:2, and 1:1 were prepared and named P1, P2, P3, P4, and P5. In addition, another PVDF solution with a concentration of 20% was spun separately under the same conditions, and the obtained control group was named P0. During the appeal process, the stirring speed was 300 r/min.

After removing the air bubbles, the prepared spinning solutions were placed in a 10 mL syringe. A needle with an inner diameter of 0.75 mm and an outer diameter of 1 mm was chosen as the spinning needle, and aluminum foil was attached to the drum receiver The voltage of electrospinning was set to 15 kV, the perfusion speed was set to 2 mL/h, the perfusion volume was set to 10 mL, the drum speed was set to 400 r/min, and the spinning distance was set to 12 cm. After spinning, the prepared PVDF/PDMS electrospinning membrane was placed in an oven to dry for use.

### 2.3. Preparation of Water-in-Oil Emulsion

In the water-in-oil emulsion, n-hexane and deionized water were chosen as the oil and water, and span 80 was selected as the active agent. For the convenience of observation, n-hexane was stained with oil red, and deionized water was stained with methylene blue. The specific operation was to mix 1 mL of deionized water and 0.1 mL of span 80 into 100 mL of n-hexane and vigorously stir at a speed of 10,000 r/min for 30 min to obtain a uniform water-in-oil emulsion.

### 2.4. Characterization

A scanning electron microscope (SEM) (Phenom XL, Phenom Scientific, Eindhoven, Noord-Brabant, The Netherlands) was used to observe the microstructure and elemental distribution of the membranes. Before observation, all samples were sprayed with gold. Fourier transform infrared spectral analysis (FTIR) (Nicolet iN10 MX, Thermo Fisher Scientifc, Waltham, MA, USA) was performed in the wavenumber region of 500–4000 cm^−1^ with a resolution of 4 cm^−1^. The wettability of the membranes was characterized by a contact angle tester (XG-CAM, XYCXIE, Shanghai, China) with a droplet size of 2 μL. A texture analyzer (TMS-Pilot, FTC, Leesburg, VA, USA) was used to characterize the mechanical properties, and the membranes were stretched at a speed of 2 mm/min. The transmittance of light was characterized by an ultraviolet-visible spectrophotometer (T6-1650F, PERSEE, Beijing, China). A polarizing microscope (CX40P, Sunny Instruments, Ningbo, China) was used to observe before and after the separation of the water-in-oil emulsion. A trace moisture analyzer (DHS-20A, LICHEN, Shanghai, China) was used to measure the water concentration of the water-in-oil emulsion before and after separation. The oil–water separation efficiency of the membrane was calculated by Equation (1):

(1) $$S=\left(1-\frac{C_{p}}{C_{f}}\right)\times 100\%$$

where S (%) is the oil–water separation efficiency, and $C_{f}$ (ppm) and $C_{p}$ (ppm) are the water concentrations in the water-in-oil emulsion before and after separation. The permeation flux of the emulsion is calculated by Equation (2):

(2) $$P=\frac{V}{A\times t}$$

where P (L m^−2^ h^−1^) is the permeation flux of the emulsion, V (L) is the total volume of the emulsion passing through the membrane, A (m^2^) is the effective area of the membrane, and t (h) is the time taken for the emulsion to pass through the membrane. The Ultraviolet (UV) protection factor is calculated by Equation (3):

(3) $$UPF=\frac{100}{T}$$

where UPF is the UV protection factor and T is the UV transmittance.

## 3. Results and Discussion

### 3.1. Morphology and Chemical Composition

**Figure 2:**
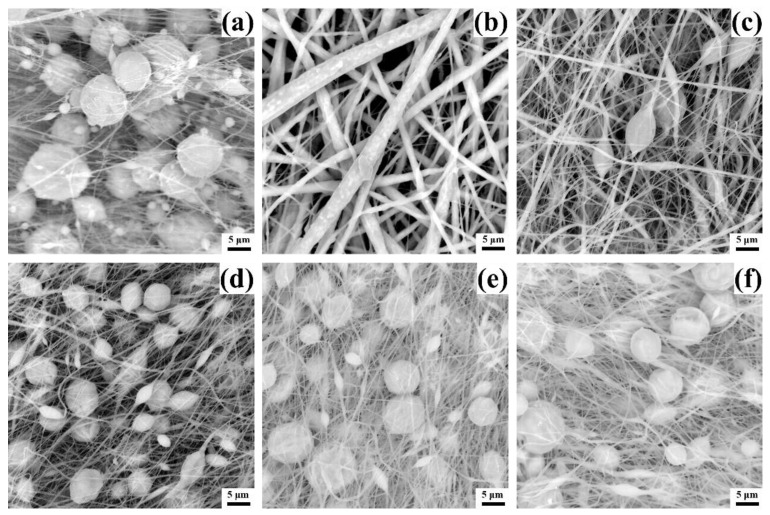
SEM images of different PVDF/PDMS electrospun membranes. (**a**) Micromorphology of P0. (**b**) Micromorphology of P1. (**c**) Micromorphology of P2. (**d**) Micromorphology of P3. (**e**) Micromorphology of P4. (**f**) Micromorphology of P5.

The micromorphology largely affects the performance of the membrane, so the microstructure of different membranes was characterized by SEM, as shown in Figure 2. It is known from the literature that M_w_ of 400,000 is sufficient to support PVDF to form fibers during electrospinning [38], and PVDF with a concentration of 20% can form fibers without microspheres [39]. However, Figure 2a shows a different result, only a small amount of fibers are formed in the pure PVDF membrane P0, and most of the PVDF exists in the form of microspheres. This phenomenon is related to the boiling point of the solvent. Most PVDF electrospinning uses a mixed solvent of acetone (boiling point 56.5 °C) and DMF (boiling point 153 °C), while Figure 2a only uses DMF as a single solvent. The addition of acetone can significantly reduce the boiling point of the mixed solvent so that the solvent can be fully volatilized during electrospinning. However, the single solvent of DMF can easily form fibers containing microspheres due to its high boiling point. Furthermore, since PDMS alone cannot be electrospun, and pure PDMS solutions cannot even be deposited on the receiver under the electric field force, no images of pure PDMS membranes are shown. However, when the PDMS solution is blended with the PVDF solution, all PDMS/PVDF membranes effectively form fibers under the same electrospinning conditions, benefiting from the change of solvent and solute. The mixture of PDMS solvent THF (boiling point 66 °C) and DMF forms a mixed solvent with a lower boiling point, which provides favorable conditions for forming fibers. Meanwhile, the addition of PVDF provides feasibility for the electrospinning of PDMS. 

In Figure 2b, membrane P1 has the thickest fiber diameter. Since the solution concentration of the added PVDF at this time is the largest, the viscosity of the spinning solution is the largest. The spinning solution’s high viscosity hinders the fibers’ stretching under the force of the electric field, increasing the fiber diameter. As the concentration of the added PVDF solution decreases, the fibers become thinner gradually, as shown in Figure 2c. When the concentration is reduced to 20%, the membrane fibers become significantly thinner, and many microspheres appear, as shown in Figure 2d. This is because when the spinning solution’s viscosity decreases, the polymer’s surface tension gradually plays a dominant role, so the polymer does not easily form fibers, and more microspheres will be created. As the concentration decreased, the diameter of the fibers did not change significantly, but the microspheres became larger, as shown in Figure 2e,f. Therefore, from the results, adding PVDF solution at a concentration of 20% is critical to ensure the production of thin fibers while reducing the size of the microspheres.

**Figure 3:**
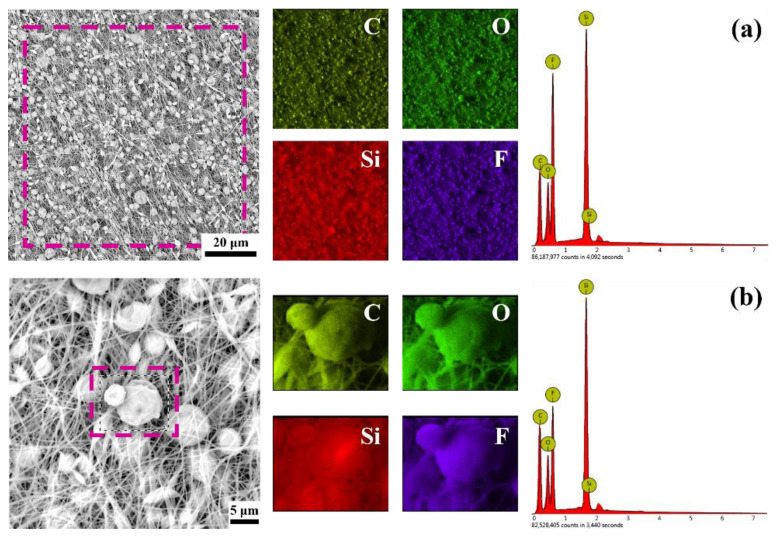
EDS analysis of membrane P3. (**a**) EDS analysis of the entire membrane. (**b**) EDS analysis of the microspheres.

To explore the specific composition and distribution of PDMS and PVDF on the membrane, EDS analysis was performed on membrane P3, as shown in Figure 3. Among them, silicon element only exists in PDMS, and fluorine element exists only in PVDF. It can be seen from Figure 3a that, on the whole, silicon (red) and fluorine (purple) are uniformly distributed on the whole membrane and show higher peaks, which proves that PDMS and PVDF are uniformly distributed over the entire membrane. This indicates that PDMS and PVDF are fully dispersed in the solution and do not separate from each other, indicating that the mixed solvent formed by DMF and THF is suitable for PDMS and PVDF. The direction of the fibers can be observed in the EDS image of fluorine, but the fibers appear blurry in the EDS image of silicon, which seems to be more concentrated on the microspheres.

EDS analysis was performed to determine the microspheres’ composition, as shown in Figure 3b. Silicon and fluorine elements can also be observed on the surface of the microspheres. However, at this time, silicon shows a higher brightness than fluorine, indicating that the proportion of PDMS on the microspheres has increased, which can also be seen from the significantly lower peak of fluorine. The phenomenon that PDMS is more likely to aggregate in the microspheres might be related to the properties of the solvent and PDMS. It should be mentioned that PDMS is only soluble in THF, not in DMF, and pure PDMS has no spinnability. Therefore, when PDMS molecular chains are cross-linked and entangled with PVDF molecular chains, the regions with higher PVDF content are more likely to form fibers. In comparison, the areas with higher PDMS content are more likely to form microspheres. In addition, THF is a volatile solvent, which will cause regions with high PDMS content to be detached from the solvent earlier in the distance from the needle to the receiver and entrain part of the PVDF to form microspheres.

**Figure 4:**
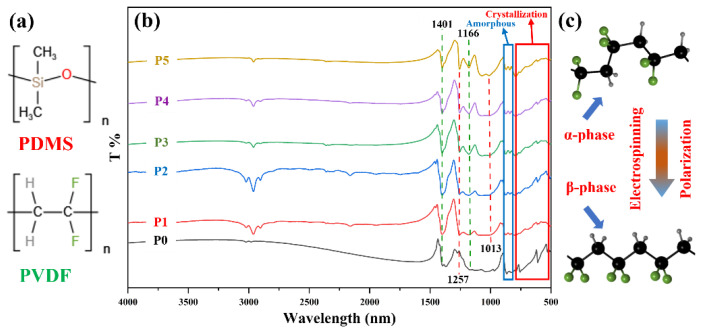
FTIR of different membranes and the principle of PVDF phase transition. (**a**) The structural formulae of PDMS and PVDF. (**b**) FTIR of the membrane. (**c**) The principle of PVDF transformation from *α* phase to *β* phase under the high voltage polarization of electrospinning.

To further verify the composition of the membrane, FTIR analysis of the membrane was performed, as shown in Figure 4. Figure 4a is the structural formula of PDMS and PVDF, which can visually display the bonds in PDMS and PVDF. In Figure 4b, the membranes P0–P5 exhibit characteristic absorption peaks of PVDF at 1401 cm^−1^ and 1166 cm^−1^, which are attributed to the bending vibration of -CH_2_- and the stretching vibration of -CF_2_-. Unlike membrane P0, membranes P1–P5 show characteristic absorption peaks of PDMS at 1257 cm^−1^ and 1013 cm^−1^, indicating that PDMS exists in the membranes. Among them, the peak at 1257 cm^−1^ is the absorption peak of -Si-CH_3_, and the absorption peak at 1013 cm^−1^ is the stretching vibration of Si-O-Si. In membranes P5–P1, with the addition of PVDF, the content of PDMS gradually decreases, and the characteristic peak intensity of PDMS steadily reduces. 

The broad red square in Figure 4b is the vibrational absorption peak of the PVDF crystallization phase, the characteristic absorption peak of the non-polar *α* phase is at around 795 cm^−1^, 760 cm^−1^, 612 cm^−1^, and 531 cm^−1^. The little blue square is the amorphous absorption peaks of PVDF at about 877 cm^−1^ and 840 cm^−1^, and the absorption peak at 840 cm^−1^ is the characteristic absorption peak of the *β* phase. As shown in Figure 4b, compared with the apparent *α*-phase absorption peak of pure PVDF membrane P0, the *α*-phase characteristic absorption peaks of P1–P5 become gentler. The *β*-phase characteristic absorption peaks are slightly enhanced, and this is because the *α* phase is partially transformed into the *β* phase under the high voltage polarization of electrospinning [40]. The principle of the transformation is shown in Figure 4c. As seen from the figure, there is a big difference between the *α* and *β* phases. The molecular chains of the *α* phase are irregularly arranged, while the *β* phase is the opposite, giving them different abilities. In addition, under certain conditions, the *α* and the *β* phases can be transformed into each other. For example, in electrospinning, the CF_2_ dipoles were rearranged and faced the same side under the action of an applied electric field, transforming from the *α* phase with good mechanical properties to the *β* phase with excellent piezoelectric properties [41].

### 3.2. Wetting Behavior

**Figure 5:**
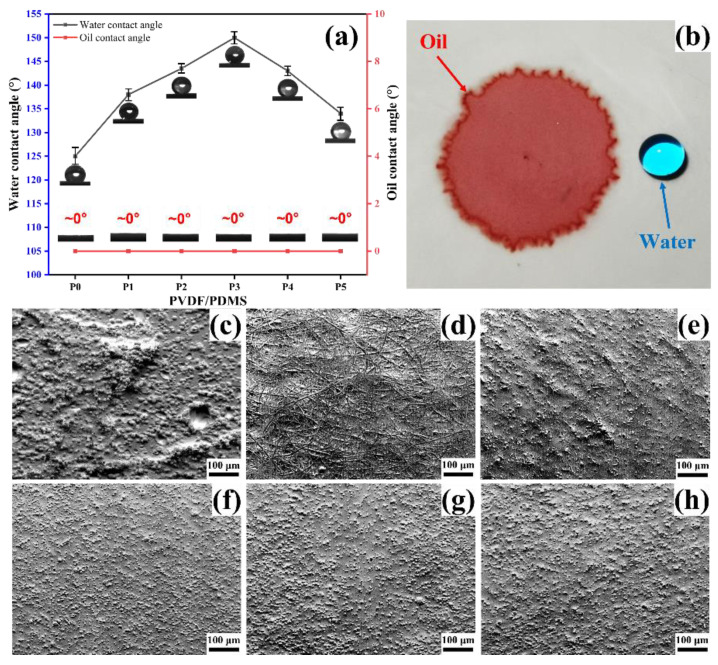
The wettability and micro-roughness of the membrane surface. (**a**) Water and oil contact angles of membranes P0–P5. (**b**) Water droplets and oil droplets showed opposite wettability on the membrane. (**c**–**h**) Micro-roughness of membrane P0–P5.

The wettability of the membrane significantly affects the separation of the oil–water mixture and the oil–water emulsion [42], so the wettability of the membranes was characterized, as shown in Figure 5. In addition, the micro-roughness is closely related to wettability [43]. To explore the relationship between the micro-roughness and the contact angle, the micro-roughness of the membrane surface was simulated by the topographical mode of SEM. As seen in Figure 5a, all the membranes have large water contact angles, and oil contact angles are almost zero. The diametrically opposite wettability allows oil droplets to diffuse in the membrane while water droplets remain spherical, as shown in Figure 5b. Furthermore, pure PVDF membrane P0 shows a lower water contact angle in Figure 5a compared with PDMS-containing membranes P1–P5, which is related to the non-uniformity of micro-roughness. It can be seen from Figure 5c that the uniformity of the surface micro-roughness of the membrane P0 is inferior to that of the PDMS-containing membrane. 

In addition, the Cassie model can explain the variation of the water contact angle of membranes P1–P5 [19]. According to the Cassie model, within a specific range, the smaller the proportion coefficient (f) of the solid–liquid contact area in the total contact area, the greater the water contact angle of the material surface. For PDMS/PVDF electrospun membranes, the value of f is related to the diameter of the fibers and the size of the microspheres. Simply put, the finer and denser the fibers, and the smaller and denser the microspheres, the larger the water contact angle of the membrane. It can be seen from Figure 5f–h that with the increase of PVDF addition, the microspheres gradually become smaller and denser, which will directly decrease the value of f. The decrease in the f value causes the water contact angle to rise, and finally, the maximum water contact angle is obtained in membrane P3. At this time, further increasing the addition of PVDF will lead to an increase in the diameter of the fibers, and traces of fibers can be observed in Figure 5e. Thickening of the fibers will increase the f value, resulting in a decrease in the water contact angle. If the added amount of PVDF is further increased, it can be found in Figure 5d that the microspheres disappear utterly, and the whole surface of the membrane is composed of thick and loose fibers. In this state, the value of f will be further increased, that is, the water contact angle will be further reduced.

**Figure 6:**
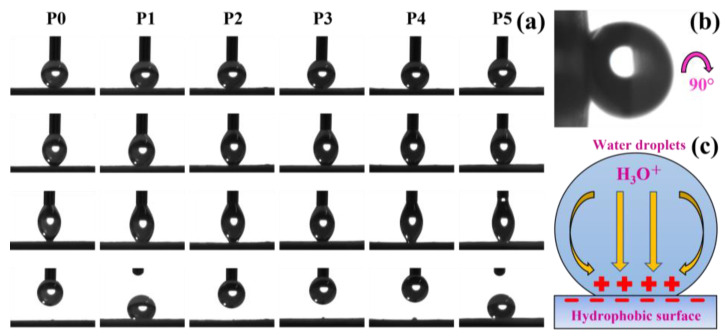
Adhesion on the surface of membranes P0–P5 and its mechanism. (**a**) Different adhesion of membranes P0–P5 to water droplets. (**b**) Water droplets still adhered to the membrane P3 surface even when the membrane was rotated by 90°. (**c**) Electrostatic interaction between the hydrophobic material and the water droplet.

In addition, the adhesion behavior of the membranes was also characterized, as shown in Figure 6a. Compared with the membrane P0, the PDMS-containing membranes P1–P5 show a greater degree of water droplet deformation and exhibit higher adhesion, which is related to the high adhesion of PDMS. In P1–P5, the membranes P1 and P5 can adhere the water droplets from the capillary. Membranes P2, P3, and P4 are the opposite, showing relatively low adhesion, but a portion of the droplet is still observed to remain on the membrane. Membrane P1 is more likely to adhere to water droplets due to the larger volume of microspheres with higher PDMS content, while membrane P1 is due to the large capillary force caused by the large capillary pores. 

An interesting phenomenon was also found during the experiment; although the membrane was rotated by 90°, the water droplets were still tightly adsorbed on the membrane P3, as shown in Figure 6b. This phenomenon can be attributed to capillary action and electrostatic interaction on the membrane surface. The high porosity of electrospinning membranes can generate large capillary forces so that droplets are adsorbed on the membranes by capillary action. However, similarly, capillary action can also facilitate the passage of oil. Furthermore, the high adhesion might also be related to the electrostatic interaction between the membrane and the droplets. Studies have found that hydrophobic materials are negatively charged even in the air. When the droplet contacts the hydrophobic surface, the cations are distributed to the solid–liquid interface to form an electrical double layer [44]. Water has high permittivity, and when the water droplets are in contact with the hydrophobic PDMS/PVDF membrane, the H_3_O^+^ in the water droplets will migrate to the solid–liquid interface, resulting in the water droplets being firmly adsorbed on the surface of the membranes under electrostatic action, as shown in Figure 6c.

### 3.3. Mechanical Properties and UV Resistance

Mechanical properties are one of the critical indicators for evaluating the performance of membranes. The fracture strength and elongation of membranes P0–P5 are recorded in Table 1. It can be seen that the membrane P0 exhibits lower elongation and fracture strength due to the discontinuous, loose, and inhomogeneous of its fibers and microspheres (Figure 5c). It should be noted that PDMS membranes generally have lower fracture strength due to their properties [24]. After adding PVDF, benefiting from the good mechanical properties of PVDF, the fracture strength of the PDMS/PVDF electrospinning membrane is improved up to 1.84 MPa. At the same time, the presence of PDMS can reduce the force between PVDF molecular chains, reduce its crystallinity, and increase the elongation of PDMS/PVDF electrospinning membranes [2]. The table shows that the membrane P5 with the least amount of PVDF added has an elongation as high as 103.85%. 

Generally speaking, to have the properties of two polymers simultaneously, it is hoped that the blend can achieve macroscopic uniformity and microscopic phase separation. As mentioned earlier, PDMS concentrates more on microspheres, while PVDF concentrates more on fibers. This behavior provides favorable conditions for microscopic phase separation. That is, the presence of microspheres can make the PDMS/PVDF membrane have both good elongation and fracture strength. In this state, increasing the content of PVDF can significantly increase fracture strength and reduce the loss of elongation. For example, the change in elongation and fracture strength from membrane P5 to membrane P3 is in Table 1. However, when the PVDF content is 2.5 times that of PDMS, the number of microspheres decreases significantly, resulting in the reduction of microscopic phase separation. At this time, although the elongation of membrane P2 increases by about 20% compared to P3, the fracture strength decreases by about 70%. Furthermore, if the PVDF content is further increased (membrane P1), the disappearance of the microspheres leads to the complete disappearance of the microscopic phase separation. Ultimately, the membrane P1 loses both good elongation and fracture stress properties. This result indicates that a reasonable ratio of PDMS to PVDF can make PDMS/PVDF membranes have both elongation and fracture strength.

**Figure 7:**
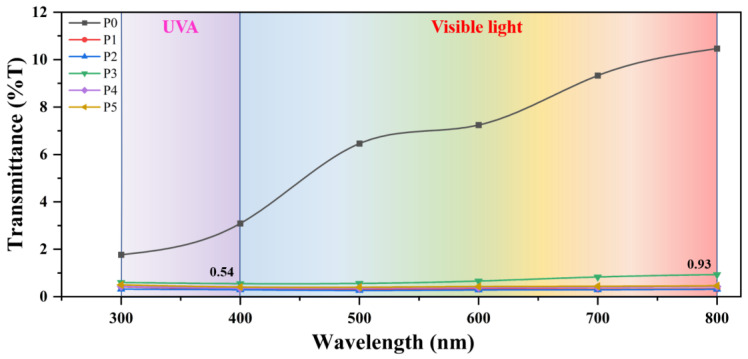
The transmittance of PDMS/PVDF membrane to ultraviolet and visible light.

In addition, PDMS has good UV resistance [45,46] and can be used outdoors for a long time. Since the bond energy of Si-O-Si is 422.5 kJ/mol, which is higher than the radiation energy of UV (314–419 kJ/mol), UV can not destroy the stability of PDMS. PVDF is also an excellent UV-resistant material, and PVDF also has good UV-shielding ability. As shown in Figure 7, the UV transmittance of pure PVDF membrane P0 is less than 3%, indicating good UV shielding ability. Furthermore, PDMS-containing membranes show lower UV transmittance, but PDMS has high UV transmittance [47]. This is because the presence of PDMS can enhance the degree of cross-linking and entanglement between PDMS and PVDF molecular chains, thereby enhancing the UV shielding ability of PDMS/PVDF membranes. It can be seen from the figure that the UV transmittance of the PDMS/PVDF electrospinning membrane is all less than 0.54%, confirming its excellent UV shielding ability. In addition, the material has a UV protection factor (UPF) of 185.2, which is much higher than the standard of UPF 50+, making it an excellent UV protection material.

### 3.4. Separation Ability of Water-in-Oil Emulsions

**Figure 8:**
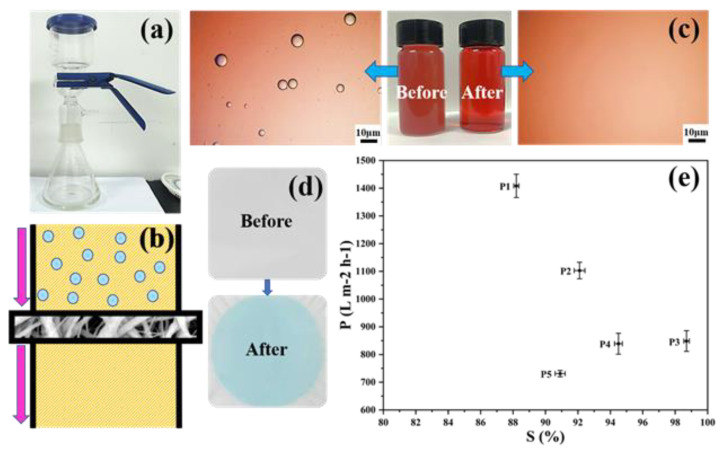
Oil–water separation capacity of membranes. (**a**) The device of the oil–water separation. (**b**) Schematic diagram of the oil–water separation. (**c**) Comparison of water-in-oil emulsions before and after separation. (**d**) Changes in the membrane surface before and after separation. (**e**) Separation efficiency and flux of membranes for water-in-oil emulsions.

The separation ability of PDMS/PVDF membranes was evaluated, as shown in Figure 8. The device and the schematic diagram of the oil-water separation are shown in Figure 8a,b. The membrane is tightly clamped in the middle of the separation device, and the water-in-oil emulsion is driven by gravity to pass through the membrane from top to bottom, realizing the selective separation of the water-in-oil emulsion. For PDMS/PVDF membranes, the oil phase can easily penetrate the material, and the membrane can continue to adsorb the oil phase under the action of capillary force brought about by high porosity. From Figure 8c, it can be seen that the emulsion after separation becomes visibly cleaner than the turbidity before separation, which can also be observed in the light micrograph. The membrane’s selective permeability to the water-in-oil emulsion is also shown in Figure 8d. Compared with the pure white membrane before separation, the membrane surface after separation is blue. The membrane’s blue color after the emulsion’s separation comes from the methylene blue used for water dyeing. In contrast, the oil red used for n-hexane dyeing does not remain on the membrane, which can be attributed to the high water contact angle and low oil contact angle of the membrane. 

Figure 8e shows the separation efficiency and flux of water-in-oil emulsions by membranes with different amounts of PVDF added. The separation efficiency of membranes P1–P5 for emulsion shows a trend of increasing first and then decreasing. The maximum separation efficiency of 98.7% is achieved in membrane P3, which is consistent with the changing direction of the contact angle, indicating that wettability is one of the critical factors affecting the oil-water separation efficiency of the membrane. In addition, with the increase in PVDF content, the flux of the membrane to the water-in-oil emulsion shows an upward trend. Benefiting from the large pore size, membrane P1 has a maximum flux of 1400 L m^−2^ h^−1^, but only has a separation efficiency of 88%. Compared with P1, P2, P4, and P5, membrane P3 can have an emulsion flux of 848 L m^−2^ h^−1^ at higher separation efficiency.

**Figure 9:**
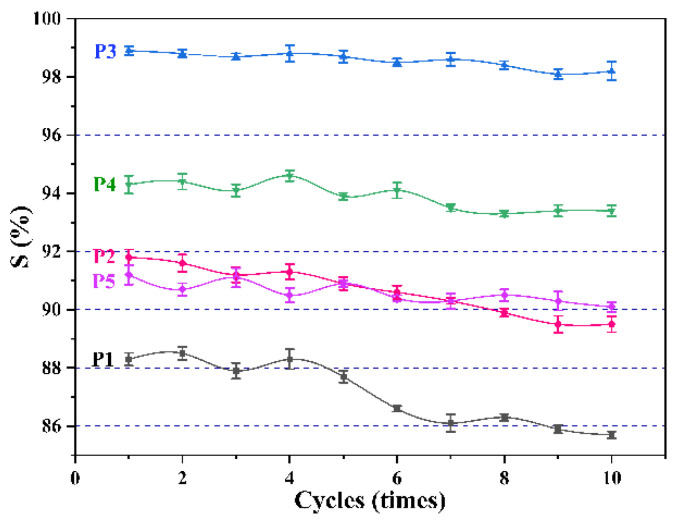
Changes in separation efficiency of different membranes over ten cycles.

In addition, the membrane’s recycling performance is also a critical indicator. Here, the change in separation efficiency of the membrane over ten cycles was tested, as shown in Figure 9. It should be mentioned that every 100 mL is a cycle. It can be seen that after ten cycles, the separation efficiency of all membranes decreases to varying degrees. As the separation progressed, tiny water droplets in the water-in-oil emulsion gradually accumulated on the membrane surface. When the amount of accumulation reaches a specific value, water is forced to pass through the pores under pressure, resulting in a decrease in the separation efficiency of the membrane. Among them, the separation efficiency of membranes P1 and P2 decrease most obviously, because they have larger pore sizes, which makes it easier for water to pass.

## 4. Conclusions

In this work, PDMS/PVDF membranes for the separation of water-in-oil emulsions were prepared by electrospinning technology, and the effects of different addition amounts of PVDF on the morphology and performance of membranes were explored. The study finds that PDMS and PVDF have a complementary relationship in electrospinning, PVDF can help PDMS to form fibers, and PDMS in turn can increase the amount of PVDF to form fibers. In addition, the membranes with microspheres show better hydrophobicity than those without microspheres, which benefited from the microspheres’ micro-roughness. The results also show that the content of PDMS in the microspheres is higher, bringing lower surface energy and better lipophilicity to the membrane surface. Interestingly, all the membranes show higher adhesion, which might be related to capillary and electrostatic interactions on the membrane surface. After comparison, when the PVDF and PDMS mass ratio reaches 2:1, the membrane shows the best all-around performance. At this ratio, the water contact angle of the membranes can reach 150°, and the oil contact angle is about 0°, showing opposite wettability. In addition, the membrane has a separation efficiency of 98.7% for water-in-oil emulsions and a flux of 848 L m^−2^ h^−1^, making it a suitable candidate for oil–water separation materials. At the same time, the membrane also shows excellent ultraviolet resistance ability, and the transmittance to ultraviolet light is lower than 0.54%, which is also a potential ultraviolet protection material.

## Figures and Tables

**Table 1:** Mechanical properties of PDMS/PVDF membranes.

	Max Pulling Force (N)	Elongation (%)	Fracture Strength (MPa)
P0	0.91	36.93	0.97
P1	1.75	81.18	0.54
P2	5.07	86.15	1.15
P3	2.64	68.30	1.84
P4	2.56	66.10	1.82
P5	0.48	103.85	0.38

## Data Availability

Data will be made available on request.
